# Relationships between systemic sclerosis and atherosclerosis: screening for mitochondria-related biomarkers

**DOI:** 10.3389/fgene.2024.1375331

**Published:** 2024-07-10

**Authors:** Fei Wang, Xiao Yan Lyu, Yi Ming Qin, Mei Juan Xie

**Affiliations:** ^1^ Department of Dermatology, West China Hospital, Sichuan University, Chengdu, China; ^2^ Laboratory of Dermatology, Clinical Institute of Inflammation and Immunology, Frontiers Science Center for Disease-Related Molecular Network, West China Hospital, Sichuan University, Chengdu, China

**Keywords:** systemic sclerosis, atherosclerosis, machine learning, mitochondria-related genes, immune infiltration

## Abstract

**Background:**

Patients with systemic sclerosis (SSc) are known to have higher incidence of atherosclerosis (AS). Mitochondrial injuries in SSc can cause endothelial dysfunction, leading to AS; thus, mitochondria appear to be hubs linking SSc to AS. This study aimed to identify the mitochondria-related biomarkers of SSc and AS.

**Methods:**

We identified common differentially expressed genes (DEGs) in the SSc (GSE58095) and AS (GSE100927) datasets of the Gene Expression Omnibus (GEO) database. Considering the intersection between genes with identical expression trends and mitochondrial genes, we used the least absolute shrinkage and selection operator (LASSO) as well as random forest (RF) algorithms to identify four mitochondria-related hub genes. Diagnostic nomograms were then constructed to predict the likelihood of SSc and AS. Next, we used the CIBERSORT algorithm to evaluate immune infiltration in both disorders, predicted the transcription factors for the hub genes, and validated these genes for the two datasets.

**Results:**

A total of 112 genes and 13 mitochondria-related genes were identified; these genes were then significantly enriched for macrophage differentiation, collagen-containing extracellular matrix, collagen binding, antigen processing and presentation, leukocyte transendothelial migration, and apoptosis. Four mitochondria-related hub DEGs (IFI6, FSCN1, GAL, and SGCA) were also identified. The nomograms showed good diagnostic values for GSE58095 (area under the curve (AUC) = 0.903) and GSE100927 (AUC = 0.904). Further, memory B cells, γδT cells, M0 macrophages, and activated mast cells were significantly higher in AS, while the resting memory CD4^+^ T cells were lower and M1 macrophages were higher in SSc; all of these were closely linked to multiple immune cells. Gene set enrichment analysis (GSEA) showed that IFI6 and FSCN1 were involved in immune-related pathways in both AS and SSc; GAL and SGCA are related to mitochondrial metabolism pathways in both SSc and AS. Twenty transcription factors (TFs) were predicted, where two TFs, namely BRCA1 and PPARγ, were highly expressed in both SSc and AS.

**Conclusion:**

Four mitochondria-related biomarkers were identified in both SSc and AS, which have high diagnostic value and are associated with immune cell infiltration in both disorders. Hence, this study provides new insights into the pathological mechanisms underlying SSc and AS. The specific roles and action mechanisms of these genes require further clinical validation in SSc patients with AS.

## 1 Introduction

Systemic sclerosis (SSc) is a chronic autoimmune connective tissue disease characterized by vasculopathy, multiorgan fibrosis, and dysregulated immune functions (Pongkulkiat et al., 2022). Its mortality rate ranks first among all rheumatic diseases (Denton and Khanna, 2017). Patients with SSc have higher risks of morbidity and mortality due to cardiovascular diseases (CVDs) and have been shown to have 1.8 times increased risk of coronary artery disease. Patients with SSc and apparent cardiovascular symptoms are at increased risk of deterioration and early death from cardiovascular causes, accounting for approximately 20%–30% of all SSc deaths (Mok and Lau, 2010). Atherosclerosis (AS) is the dominant pathological cause of CVD and can significantly affect the morbidity and mortality of SSc patients (Wu et al., 2022). The incidence of AS in patients with SSc was reported to be 56% (Pagkopoulou et al., 2023) as SSc is an independent risk factor that can accelerate AS (Mok et al., 2011).

SSc and AS affect each other in clinical processes; therefore, exploring their shared molecular mechanisms is essential. Numerous studies have explored the risk factors of AS in patients with SSc. One study demonstrated that SSc patients with AS had higher modified Rodnan skin scores (mRSSs) and intima-media thicknesses (Phuong et al., 2022). Another study showed that the serum PCSK9 levels in SSc were positively correlated with carotid intima-media thickness (*p* = 0.031) (Ferraz-Amaro et al., 2020). Additionally, the levels of oxidative stress markers are higher in the sera of SSc patients. Oxidative stress is a significant risk factor in the damage of vascular endothelial cells, which leads to AS (Li et al., 2022). Mitochondria are the energy supply stations that maintain cellular homeostasis, and they are highly susceptible to oxidative stress that can lead to severe damage (Chang et al., 2023). Mitochondrial damage plays vital roles in the pathophysiology of SSc and AS, and mitochondria appear to be hubs linking SSc to AS. Mitochondrial damage activates innate immunity and facilitates the development of autoimmune diseases (Rongvaux, 2018). SSc reduces mitochondrial respiration and fusion, thereby inducing mitochondrial injury (Cantanhede et al., 2022) that causes endothelial dysfunction; these abnormal endothelial cells secrete more inflammatory cytokines and show increased oxidized low-density lipoprotein (LDL) levels associated with plaque formation (Prajapat et al., 2023). Mitochondrial injury also causes pyroptosis of the endothelial cells and hypertrophy of the smooth muscle cells (Shemiakova et al., 2020). Subsequently, more reactive oxygen species (ROS) are produced, contributing to oxidative injury of the arterial walls and arterial stiffness (Vazquez-Agra et al., 2023). The identification of common mitochondria-related biomarkers between SSc and AS is expected to help in the diagnosis and treatment of SSc combined with AS. Mitochondrial fusion and fission have critical impacts on T-cell differentiation (Jia et al., 2023). Mitochondria are the core organelles of energy metabolism in the body, and the differentiation of distinct T cell subsets relies on different metabolic pathways. Glycolysis is the main pathway of development of the Th1/Th2/Th17 cells, and fatty acid oxidation (FAO) is the main pathway of Treg development (Hu et al., 2018). These findings suggest that mitochondrial dysfunction is strongly associated with immune infiltration.

In this study, we screened the biomarkers of mitochondrial dysfunction associated with the pathogenesis of SSc and AS as well as explored the associations between mitochondria-related hub genes and immune infiltration. First, we identified the differentially expressed genes (DEGs) with common tendencies between SSc and AS; then, we considered the intersections of these DEGs with the mitochondria-related genes. Second, we predicted four mitochondria-related hub genes using two machine learning algorithms and investigated the relationships between the hub genes and immune infiltration. Finally, we predicted the transcription factors (TFs) for the four hub genes. This study hereby provides new insights into the shared pathomechanisms between SSc and AS.

## 2 Materials and methods

### 2.1 Data collection

**FIGURE 1:**
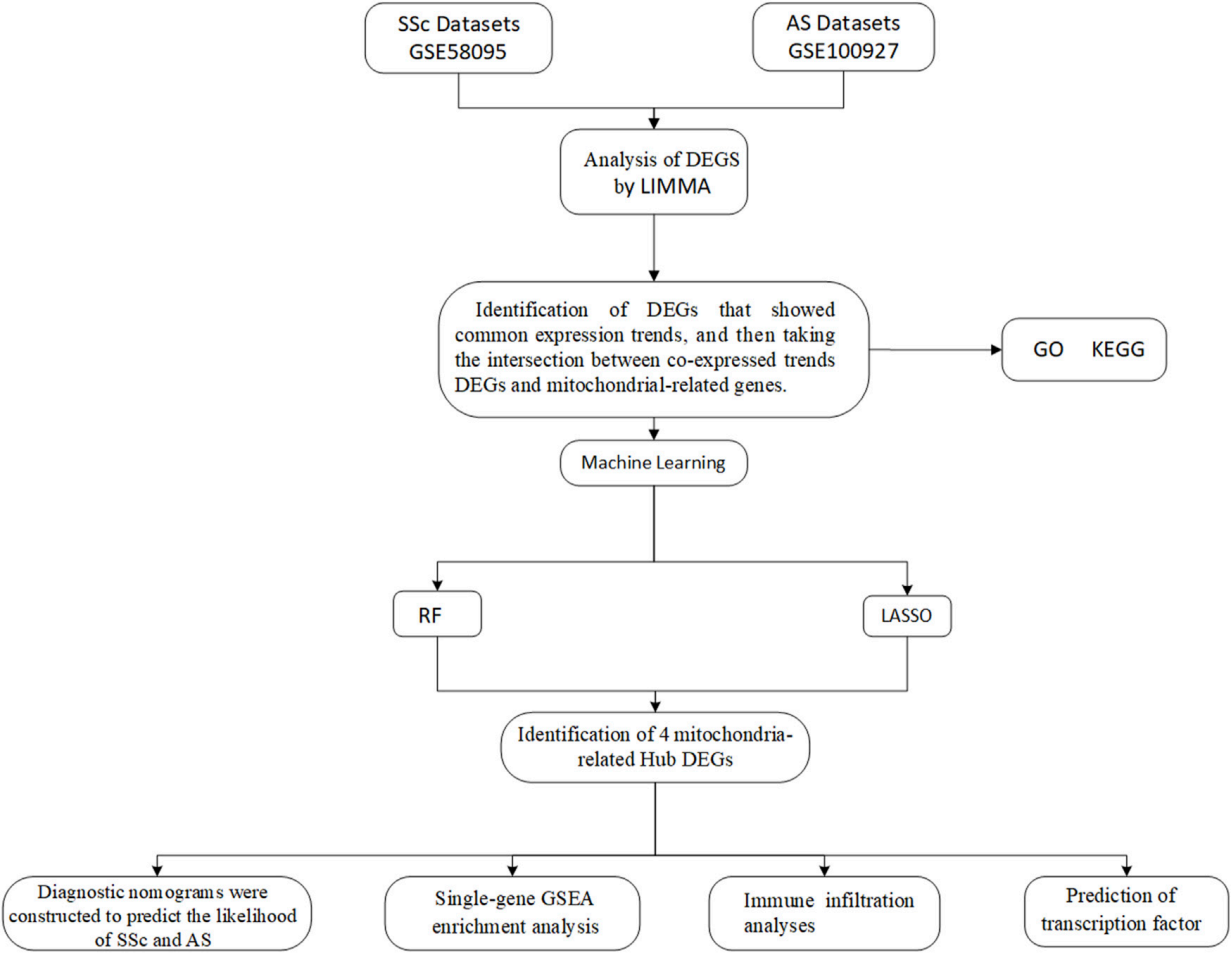
Flowchart of the study design.

We searched the Gene Expression Omnibus (GEO) database for the keywords “systemic sclerosis” and “atherosclerosis” to obtain the SSc- and AS-related datasets. The mitochondria-related genes were then retrieved from GeneCard. First, the datasets were derived from human samples. Second, each dataset had to include at least ten samples. We downloaded the GSE58095 and GSE100927 datasets separately from GPL10958 and GPL17077. The GSE58095 dataset contains skin biopsies from 49 SSc patients and 43 healthy controls. The GSE100927 dataset contains 69 arteriosclerotic plaque samples and 35 normal arterial tissue samples (Table 1). The patients with AS were predominantly male, while the patients with SSc were predominantly female. In the non-atherosclerotic group, diabetes and hypertension were present in 1.6% and 24.2% of the patients, respectively. In the AS group, diabetes and hypertension were present in 30.9% and 79.1% of the cases, respectively. Compared to the non-atherosclerotic group, the patients with AS had significantly higher incidences of hypertension and diabetes; this was also in agreement with the preexisting clinical results. There were no statistical differences between the patient and control groups in terms of age or sex. The demographic characteristics of the participants are presented in Table 1, and a flow diagram of the study is shown in Figure 1.

**TABLE 1:** Details of the GEO datasets used in this study.

Characteristic	GSE100927 (GPL17077)		GSE58095 (GPL10958)	
	Non-atherosclerotic group	AS patients	Healthy control	SSc patients
Included patients (n)	35	69	43	49
Age (years), mean (range)	64 (47–73)	68 (58–73)	46 (37–54)	52 (46–62)
Gender (F:M)	14:21	27:42	32:11	35:14
Diabetes mellitus	1.6%	30.9%	NA	NA
Hypertension	24.2%	79.1%	NA	NA

SSc, systemic sclerosis; AS, atherosclerosis.

### 2.2 Differential gene expression analysis

The DEGs of the GSE58095 and GSE100927 datasets were analyzed using the “limma” package in R software. The screening condition for the GSE58095 dataset was set at | log2FC | > 1 and adjusted to *p* < 0.05. For the GSE100927 dataset, the screening condition was set at | log2FC | > 1 and adjusted to *p* < 0.05. The results of the differentiation analyses were displayed as heat maps and volcanic graphs.

### 2.3 Identification of mitochondria-related DEGs

We identified 2,676 mitochondria-related genes by searching the GeneCard database, with a relevance score greater than 1 as the screening condition. We screened the DEGs showing common expression trends. The mitochondria-related DEGs were identified by considering the intersection between the coexpressed DEGs and mitochondria-related genes.

### 2.4 Enrichment analyses of DEGs

We performed GO and KEGG functional enrichment analyses of the screened differential genes by setting the screening condition to *p* < 0.05.

### 2.5 Identification of mitochondria-related hub DEGs

We used the least absolute shrinkage and selection operator (LASSO) and random forest (RF) methods to identify the critical prognostic genes and used the “glmnet” package in R to establish a regression model. The penalty coefficient was determined through 10 cross-validations. The RF model was constructed using the “randomForest” package in R. The top-10 variables were then screened based on their relative importance scores. The mitochondria-related hub DEGs were identified by taking the intersection between the results from the LASSO and RF algorithms through a Venn diagram.

### 2.6 Nomogram development based on diagnostic biomarkers

We constructed nomograms to evaluate the diagnostic values of the mitochondria-related hub DEGs for the two datasets using the “rms” package in R. The receiver operating characteristic (ROC) and calibration curves were then used to assess the reliability of the model predictions. The mitochondria-related hub DEGs were validated for both the GSE58095 and GSE100927 datasets.

### 2.7 Immune infiltration analyses of SSc and AS

We used the CIBERSORT tool to perform deconvolution analysis to estimate the immune infiltrations of 22 immune cell types. We further assessed the relationships between the mitochondria-related hub DEGs and these immune cells to present the results as heat maps. Gene set enrichment analysis (GSEA) was performed next to analyze the biological functions of each hub gene.

### 2.8 TF prediction

The online software NetworkAnalyst was used to construct the gene interaction network of the TFs for the mitochondria-related hub DEGs; this network was visualized using Cytoscape.

## 3 Results

### 3.1 Identification of DEGs

**FIGURE 2:**
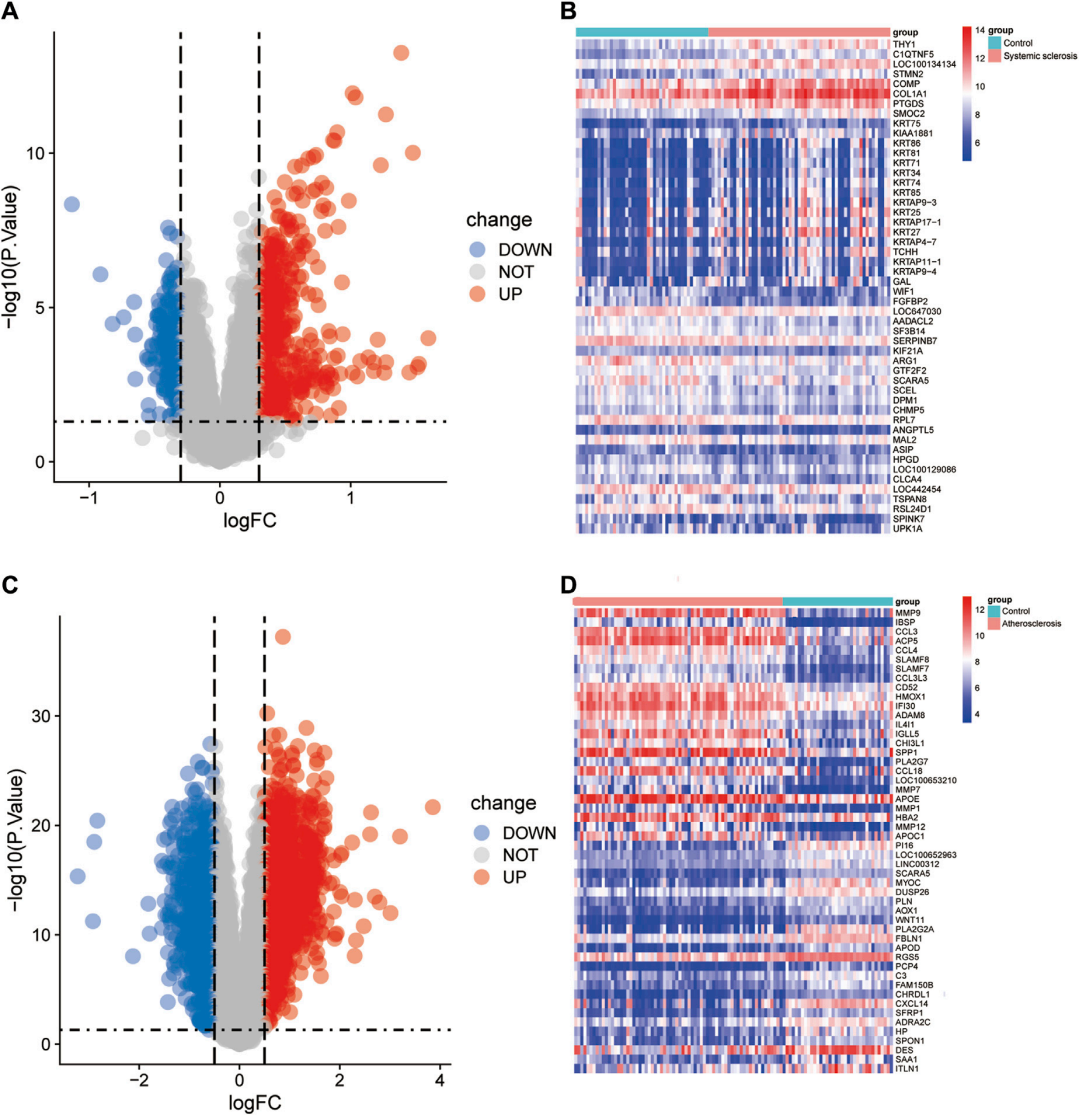
Identification of differentially expressed genes (DEGs). DEG heatmaps and volcano plots for the **(A, B)** SSc and **(C, D)** AS datasets.

**FIGURE 3:**
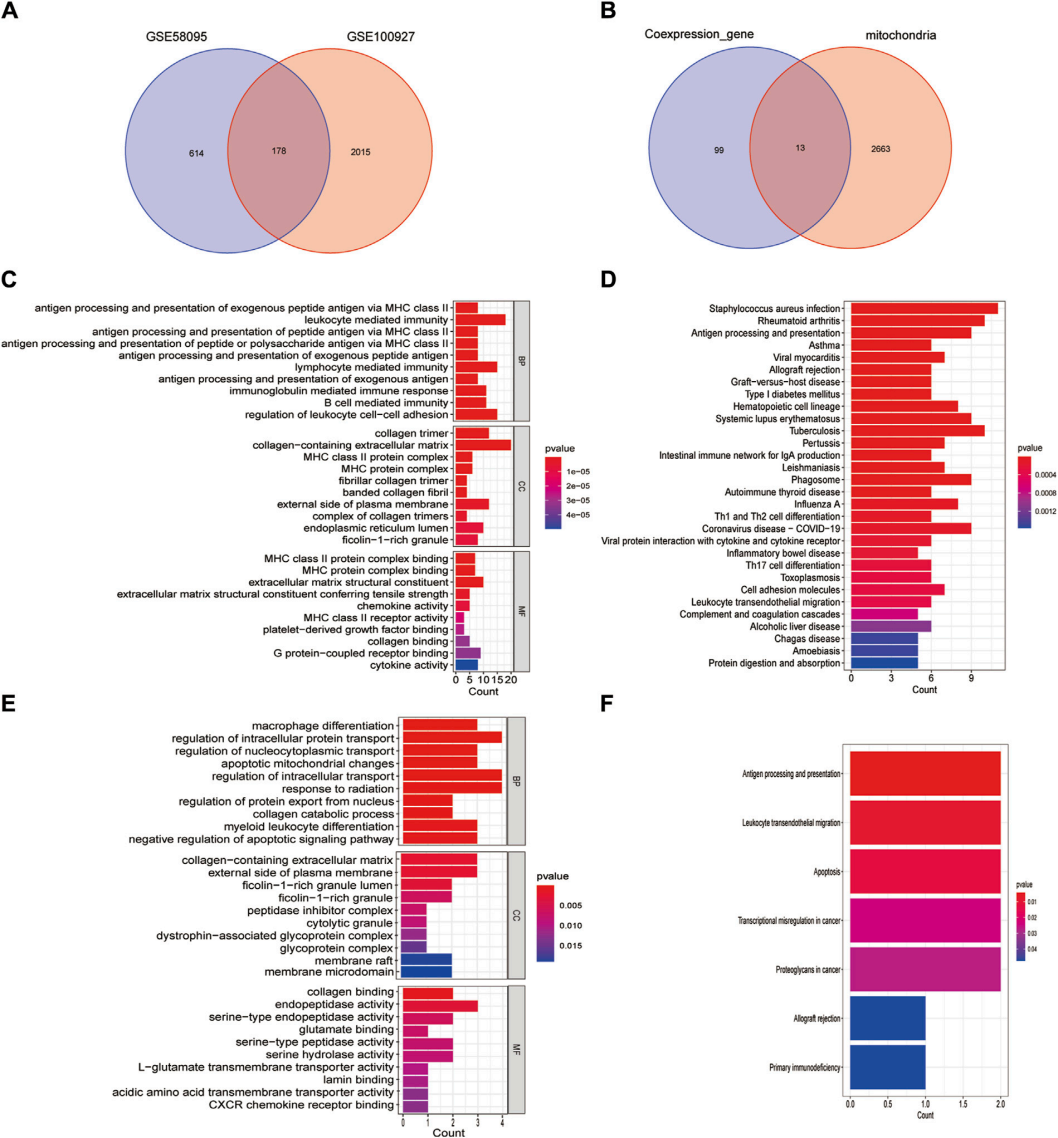
Identification of mitochondria-related DEGs and functional enrichment between SSc and AS. **(A)** Venn diagram of the DEGs; **(B)** Venn diagram of the mitochondria-related DEGs; **(C, D)** GO and KEGG enrichment analyses of DEGs with the same expression trends; **(E, F)** GO and KEGG enrichment analyses of the mitochondria-related DEGs.

A total of 792 DEGs were identified in the GSE58095 dataset, of which 504 were upregulated and 288 were downregulated; further, a total of 2,193 DEGs were identified in the GSE100927 dataset, of which 1,287 were upregulated and 905 were downregulated. The DEG expressions for the GSE58095 and GSE100927 datasets are represented via volcano maps in Figure 2A, B, where the upregulated genes are in depicted in red and downregulated genes are shown in blue. The top-50 differential genes are represented as heatmaps in Figure 2C, D. These DEGs are also shown as Venn diagrams in Figure 3A).

### 3.2 Identification of mitochondria-related DEGs

We screened the DEGs that had the same expression trends (Supplementary Table S1) and selected their intersection with the mitochondria-related genes (Figure 3B). Thirteen mitochondria-related DEGs were thus identified as IFI6, CTSB, SLC1A3, FSCN1, HCLS1, CD4, MMP9, GZMB, IFI27, CXCL12, GAL, BARD1, and SGCA. DEGs having the same expression trends were mainly enriched through antigen processing and presentation of exogenous peptide antigens via major histocompatibility complex (MHC) class II in the biological process (BP) enrichment analysis, by the collage trimer in the cellular component (CC) enrichment analysis, and by MHC class II protein complex binding in the molecular function (MF) enrichment analysis (Figure 3C); the KEGG pathway analysis revealed that these genes were enriched in *Staphylococcus aureus* infections, rheumatoid arthritis, as well as antigen processing and presentation (Figure 3D). The mitochondria-related DEGs were mainly enriched by macrophage differentiation in the BP enrichment analysis, by the collagen-containing extracellular matrix in the CC enrichment analysis, and by collagen binding in the MF enrichment analysis (Figure 3E); the KEGG pathway analysis revealed that these genes were enriched in antigen processing and presentation, leukocyte transendothelial migration, and apoptosis (Figure 3F).

### 3.3 Screening mitochondria-related hub genes and their diagnostic values

**FIGURE 4:**
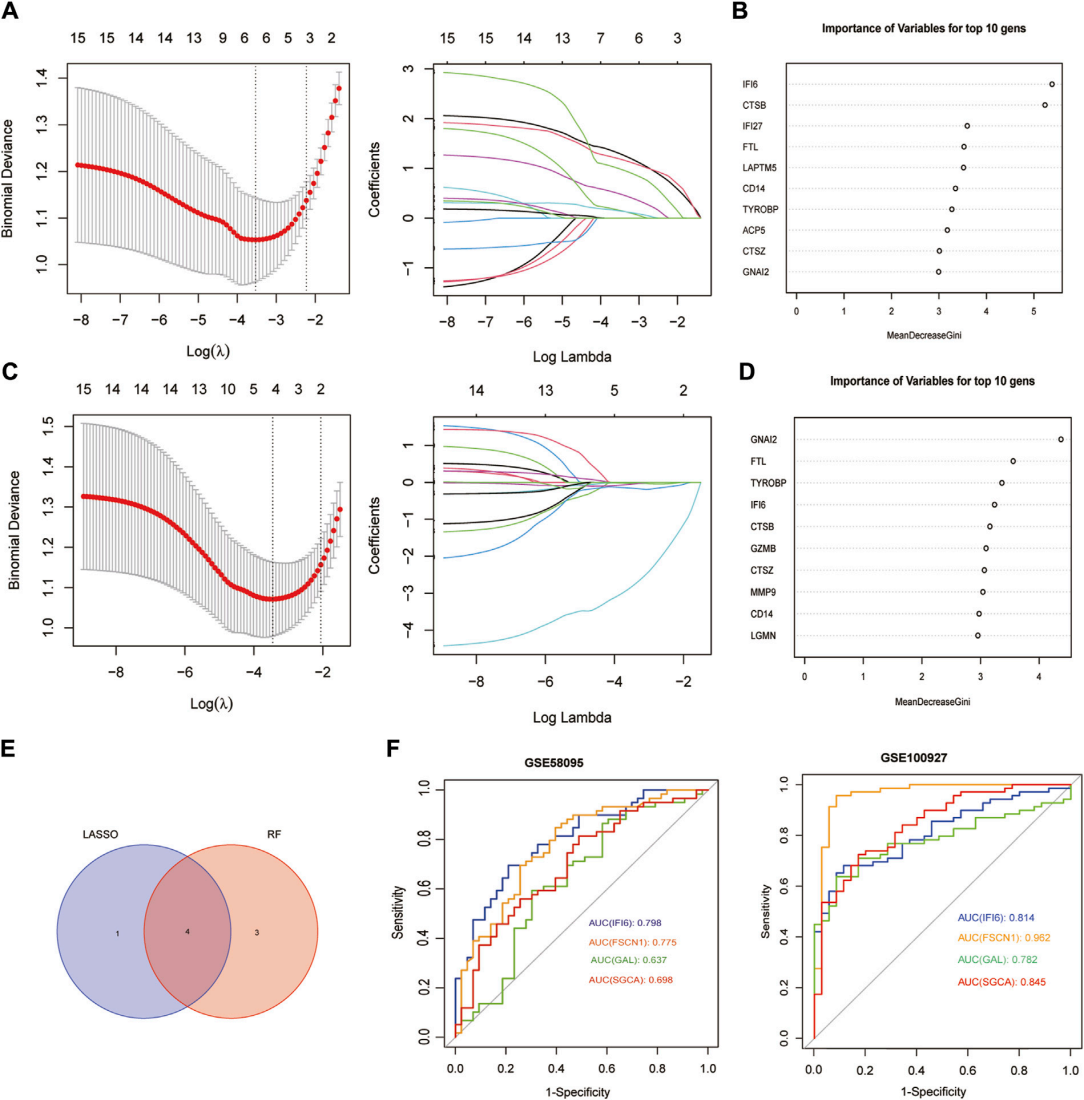
Screening of mitochondria-related hub genes and evaluation of their diagnostic values: **(A)** LASSO analysis for screening mitochondria-related hub genes in GSE58095; **(B)** identification of mitochondria-related hub genes according to the importance of variables by random forest (RF) analysis of GSE58095; **(C)** LASSO analysis for screening mitochondria-related hub genes in GSE100927; **(D)** identification of mitochondria-related hub genes according to the importance of variables by RF analysis of GSE100927; **(E)** Venn diagram of the four common mitochondria-related hub genes between the two datasets; **(F)** receiver operating characteristic (ROC) curves of the four hub genes to assess their diagnostic values in the GSE58095 and GSE100927 datasets.

**FIGURE 5:**
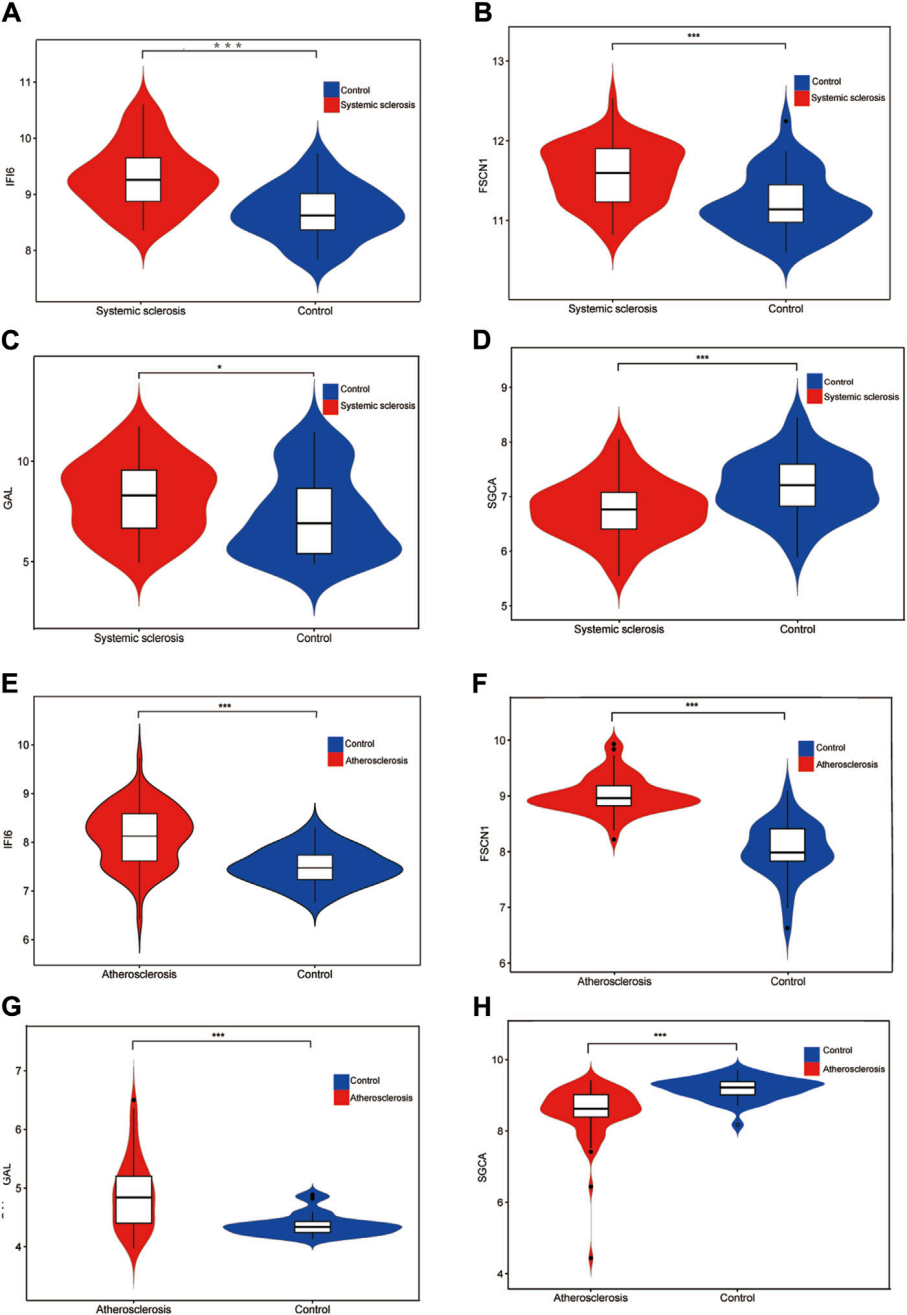
Expression levels of the four hub genes in the GSE58095 and GSE100927 datasets **(A)** Expression level of IFI6 in GSE58095; **(B)** Expression level of FSCN1 in GSE58095; **(C)** Expression level of GAL in GSE58095; **(D)** Expression level of SGCA in GSE58095; **(E)** Expression level of IFI6 in GSE100917; **(F)** Expression level of FSCN1 in GSE100927; **(G)** Expression level of GAL in GSE100927; **(H)** Expression level of SGCA in GSE100927. *p < 0.05; **p < 0.01; ***p < 0.001.

Using the LASSO algorithm, we identified five mitochondria-related genes in SSc and AS (Figure 4A, C). The top-10 genes in SSc and AS each were first identified using the RF method (Figure 4B, D), of which seven genes appeared to be coexpressed. We used the intersection of the results of these two algorithms to identify four mitochondria-related hub genes (IFI6, FSCN1, GAL, and SGCA) (Figure 4E). The ROC curves were then used to estimate the diagnostic values of these four hub genes (Figure 4F); SGCA was observed to be downregulated (Figure 5D, H), whereas IFI6, FSCN1, and GAL were noted to be upregulated in both the datasets (Figure 5A–G).

### 3.4 Nomogram development based on diagnostic biomarkers

**FIGURE 6:**
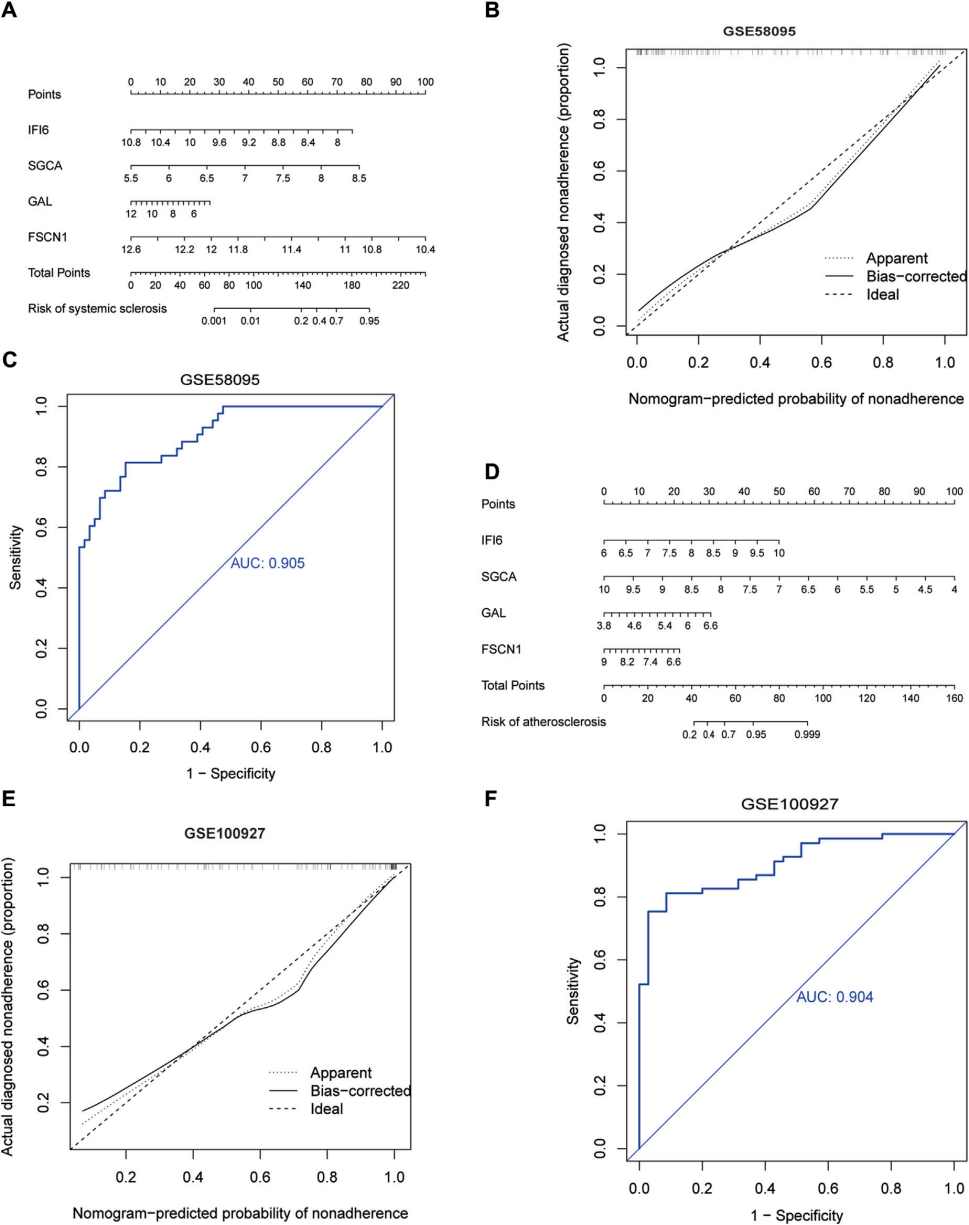
Development of the diagnostic nomogram model: **(A)** nomogram predicting the probability of SSc; **(B)** calibration curves of the SSc risk models; **(C)** ROC curve of the SSc risk model; **(D)** nomogram predicting the probability of AS; **(E)** calibration curves of the AS risk models; **(F)** ROC curve of the AS risk model.

We constructed a nomogram model that included the scores of the four hub genes (Figure 6A, D). The bias-corrected curve was close to the ideal calibration curve, indicating good calibration of the model (Figure 6B, E). The area under the curve (AUC) values for the two datasets were 0.905 and 0.904 (Figure 6C, F), indicating that the model was reliable.

### 3.5 Immune infiltration analyses

**FIGURE 7:**
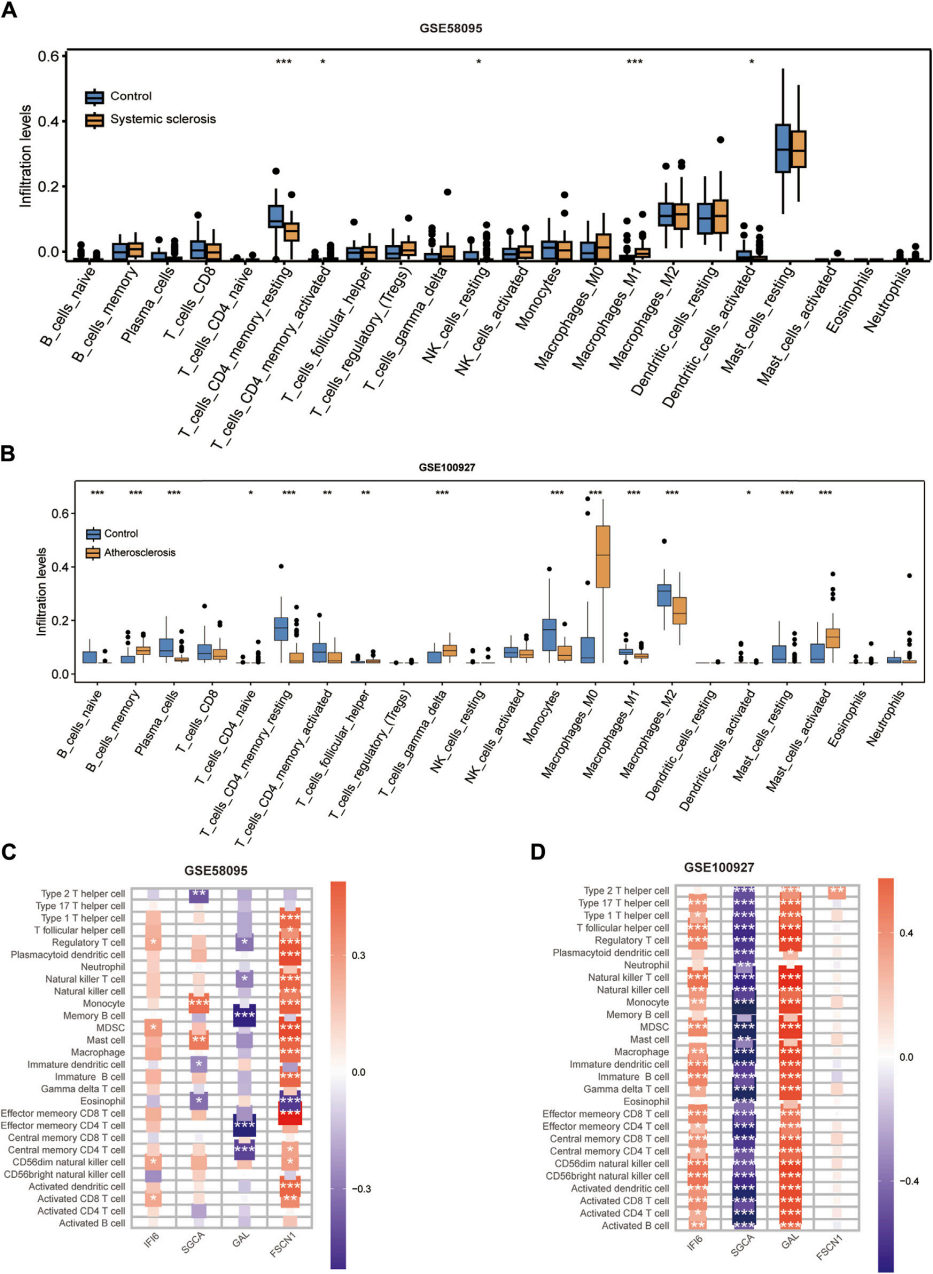
Immune cell infiltration analyses in SSc and AS: **(A)** boxplot showing the comparison of 22 kinds of immune cells between SSc and the control group; **(B)** boxplot showing the comparison of 22 kinds of immune cells between AS and the control group; **(C)** heatmap representing the associations of the differentially infiltrated immune cells with the four hub genes in SSc for the threshold of *p* < 0.05; *p < 0.05; **p < 0.01; ***p < 0.001; **(D)** heatmap representing the associations of the differentially infiltrated immune cells with the four hub genes in AS for the threshold of *p* < 0.05; *p < 0.05; **p < 0.01; ***p < 0.001.

**FIGURE 8:**
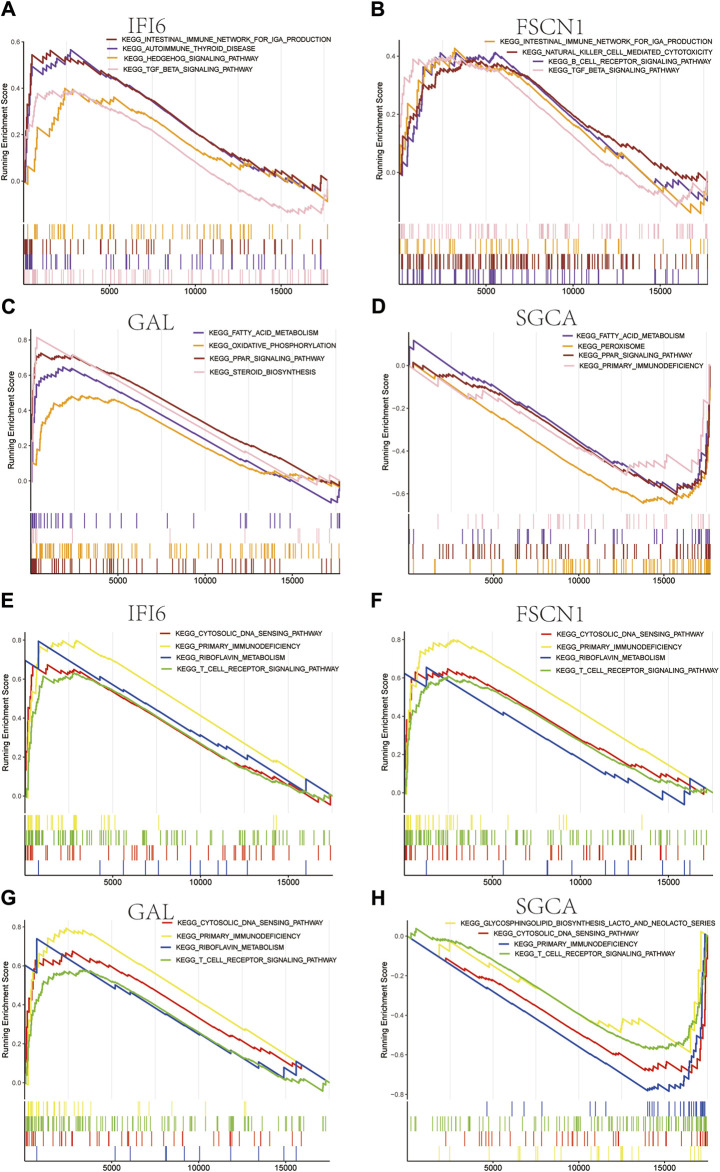
Gene set enrichment analysis (GSEA) for the four mitochondria-related hub genes in **(A–D)** SSc and **(E–H)** AS.

We used the CIBERSORT algorithm to evaluate the immune cell infiltrations between the case and control groups of both the GSE100927 and GSE58095 datasets. The AS group showed significantly elevated numbers of memory B cells, γδT cells, M0 macrophages, and activated mast cells (Figure 7A), whereas the resting memory CD4^+^ T cells and M1 macrophages was significantly elevated in patients with SSc (Figure 7B). The four hub genes were also closely linked to multiple immune cells (Figure 7C, D). Single-gene enrichment analysis showed that IFI6 and FSCN1 were enriched in the intestinal immune network of the immunoglobulin A production pathway in SSc (Figure 8A, B), whereas IFI6 and FSCN1 were enriched in the primary immunodeficiency signaling pathway in patients with AS (Figure 8E, F). GAL and SGCA were associated with fatty acid metabolism in the GSE58095 dataset (Figure 8C, D); GAL and SGCA were also enriched in the primary immunodeficiency and cytosolic DNA sensor in the GSE100927 dataset (Figure 8G, H).

### 3.6 TF prediction

We predicted a total of 20 TFs for the key genes (Supplementary Figure S1A), of which two TFs, BRCA1 and PPARγ, were highly expressed in SSc and AS (Supplementary Figure S1B–E).

## 4 Discussion

SSc mortality rates have increased significantly, and comorbidities related to CVD that is initiated by AS can result in hospitalization (Psarras et al., 2017). AS occurs in up to 56% of patients with SSc, and asymmetric dimethylarginine (ADMA) as an important mediator in vascular injury is often increased in the serum of patients with SSc and correlated with the progression of AS (Pagkopoulou et al., 2023). In addition, oxidative stress markers are increased in the serum of SSc patients and positively correlated with low-density lipoprotein cholesterol (LDL-C) levels (Ibrahim-Achi et al., 2023), and LDL-C has been recognized as a significant contributing factor to the risk of developing AS. Oxidative stress leads to severe mitochondrial damage (Chang et al., 2023), which in turn may play crucial roles in the pathophysiologies of SSc and AS. This study aimed to identify the mitochondria-related hub genes of SSc and AS.

In this study, we identified 112 genes that had the same tendencies in both disorders; of these, 13 mitochondria-related genes were identified as follows: IFI6, CTSB, SLC1A3, FSCN1, HCLS1, CD4, MMP9, GZMB, IFI27, CXCL12, GAL, BARD1, and SGCA. GO and KEGG enrichment analyses showed that these genes were significantly enriched in macrophage differentiation, collagen-containing extracellular matrix, collagen binding, antigen processing and presentation, leukocyte transendothelial migration, and apoptosis. This suggests that two points need to be considered: first, macrophage polarization, collagen synthesis, and abnormal immune conditions were common pathogenic mechanisms in these two diseases; second, impaired mitochondrial function may have affected these processes.

We used two machine learning algorithms to screen four mitochondria-related hub genes, namely IFI6, FSCN1, GAL, and SGCA. Interferon-alpha-inducible protein 6 (IFI6) is an interferon (IFN)-stimulated protein that is enriched in the inner mitochondrial membrane (Cheriyath et al., 2018); IFI6 is highly expressed in the microvascular endothelial cells (MVECs) of SSc patients with interstitial lung disease (Piera-Velazquez et al., 2021), indicating that IFI6 is involved in the development of SSc vasculopathy. IFI6 is also vital for innate immunity against multiple viruses (Dukhovny et al., 2019) as viral infections can drive the onset of SSc in susceptible individuals (Fineschi, 2021; Chen et al., 2023). Chronic inflammation resulting from viral infections promotes AS development (Poznyak et al., 2020); IFI6 is associated with the type I interferon-signaling pathway, and these type I interferons in plaques are known to aggravate AS; additionally, type I interferons are essential mediators of the pathogenesis of AS (Jia et al., 2017; Chen et al., 2020; Mathian et al., 2023).

Fascin actin-binding protein 1 (FSCN1) is a member of the fascin family (Ou et al., 2020) and contributes to mitochondrial actin filament remodeling to promote mitochondrial oxidative phosphorylation (Lin et al., 2019). FSCN1 is an endothelial activation marker, and elevated levels of FSCN1 often imply vascular damage (Bai et al., 2023). No relevant studies have explored the relationships between FSCN1 and SSc; however, its expression is associated with epithelial–mesenchymal transitions (EMTs) in other fibrotic diseases, where the EMT process can be reversed by inhibiting such expression (Fu et al., 2020). EMT occurs during skin fibrosis and includes fibroblast–myofibroblast transitions (FMTs) (Nikitorowicz-Buniak et al., 2015). EMT also promotes atherosclerotic plaque formation by linking inflammation with tissue remodeling (Peng et al., 2022). In summary, inhibition of EMT is a significant concern in the treatment of SSc and AS, and the relationships between EMT and FSCN1 expressions in SSc and AS need to be explored further. SGCA-null mice are more prone to develop muscle fibrosis.

Glutamic acid meta-galanin (GAL) is a neuropeptide that maintains the homeostasis of energy metabolism and is known to induce mitochondrial damage in several diseases. The ATP levels and mitochondrial membrane potential were reported to decrease when the cochlear basilar membrane was treated with GAL (Guo et al., 2020). GAL-induced cognitive impairment has been noted to result in mitochondrial injury (Gong and Jia, 2023). Serum GAL-3 levels are significantly elevated in patients with SSc and show good diagnostic value (AUC = 0.903) (Gruszewska et al., 2020). In SSc patients, GAL-9 expression is elevated in both the skin and serum and is associated with increased mortality as well as organ involvement (Chihara et al., 2018). GAL-9 is also related to immune imbalances and known to promote fibrosis by inhibiting Th1 production of interferon-gamma (Saigusa et al., 2017). Studies on the association between GAL and AS have mainly focused on GAL-3 and GAL-9; the formation of plaques in GAL-9-deficient mice is significantly reduced after a high-fat diet (Krautter et al., 2022), and GAL-9 can repolarize macrophages into the proinflammatory phenotype to accelerate plaque formation. GAL-3 is known to induce vascular calcification via MAPK signaling (Tian et al., 2022). GAL-3 and GAL-9 are involved in disease development; however, there are no studies investigating the action mechanisms of GAL-3 and GAL-9 in SSc comorbid with AS. The α-sarcoglycan gene (SGCA) is a protein-coding gene; in the present study, SGCA expression was downregulated in both SSc and AS. SGCA is critical for cell membrane stability and linking between the actin-based cytoskeleton and extracellular matrix. As a dystrophin-associated glycoprotein, SGCA influences the biology of vascular smooth muscles (Kaplan and Morgan, 2022); smooth muscle cells play crucial roles in the development of AS (Zhang et al., 2023). In SSc, abnormalities of the vascular smooth muscle are always considered to be associated with vascular dysfunction (Potjewijd et al., 2023). We speculate that SGCA is functional in the vascular damage of SSc patients and promotes AS progression.

Similar to previous findings, the memory B cells, γδT cells, M0 macrophages, and activated mast cells were significantly elevated in AS (Xiong et al., 2022). Switched memory cells can express IgG antibodies, and IgG levels are linked to AS progression (Tsimikas et al., 2007). The γδT cells promote early atherosclerotic lesion formation and plaque instability (Gil-Pulido et al., 2022). Mast cells promote the recruitment of neutrophils and production of extracellular traps to aggravate inflammatory responses, resulting in plaque rupture and thrombosis (Elieh-Ali-Komi et al., 2024). This study showed that resting memory CD4^+^ T cells were significantly decreased in SSc lesions similar to previous findings (Tu et al., 2022). M1 macrophages exert proinflammatory effects, and several studies have indicated that M1 macrophage polarization may aggravate inflammation in SSc and promote skin fibrosis (Park et al., 2021; Zhang et al., 2022). GSEA showed that IFI6 and FSCN1 were associated with immune-related pathways in both AS and SSc, while GAL and SGCA were related to mitochondrial metabolism pathways in both SSc and AS. GAL and SGCA may participate in fatty acid metabolism in SSc, and such disturbances in the fatty acid metabolism can cause mitochondrial dysfunction (Noels et al., 2021); these two genes are also involved in cytosolic DNA sensing in AS and can detect the cytosolic DNA to activate downstream interferon signaling (Li et al., 2013). Cyclic GMP-AMP synthase (cGAS) is a cytosolic DNA sensor, and STING is a downstream protein of cGAS; the STING pathway is activated in AS and is associated with plaque instability (Wan et al., 2022; Ueda et al., 2023). However, no studies have linked the STING pathway or AS to GAL or SGCA. The present study also predicted and validated the associated TFs using two datasets; the TFs BRCA1 and PPARγ were highly expressed in both datasets and are related to the IFI6 gene.

Mitochondrial dysfunction can damage endothelial function, contributing to AS development in SSc patients (Prajapat et al., 2023). However, there are no studies on the role of mitochondrial-metabolism-related genes in the association between these two diseases. The present study is the first to utilize machine learning algorithms to identify mitochondria-related hub genes and reveal a potential link between SSc and AS.

Although the findings of this study are novel and encouraging, there are some inevitable limitations. We performed bioinformatics analysis without *in vitro* or *in vivo* experiments to validate the predicted results. To better understand the common pathogenic molecular mechanisms of SSc and AS, subsequent cell or animal experiments are required on the predicted results to verify the conclusions; this is expected to provide ideas for the clinical diagnosis and treatment of SSc patients with AS.

## 5 Conclusion

We identified four common mitochondria-related hub genes (IFI6, FSCN1, GAL, and SGCA) between SSc and AS as well as showed that these genes had high diagnostic values. IFI6 and FSCN1 are involved in immune-related pathways in both disorders, while GAL and SGCA are related to the mitochondrial metabolism pathways in both disorders. This study provides new insights into the common pathological mechanisms underlying SSc and AS. However, further clinical studies are required to validate these roles and action mechanisms of these genes in patients with SSc and AS.

## Data Availability

The original contributions presented in the study are included in the article/Supplementary Material, and any further inquiries may be directed to the corresponding author.

## Abbreviations

SSc: systemic sclerosis; AS: atherosclerosis; GEO: Gene Expression Omnibus; LASSO: least absolute shrinkage and selection operator; RF: random forest; CVD: cardiovascular disease; mRSS: modified Rodnan skin score; ROS: reactive oxygen species; FAO: fatty acid oxidation; TF: transcription factor; DEG: differentially expressed gene; BP: biological process; CC: cellular component; MF: molecular function; ADMA: asymmetric dimethylarginine; LDL-C: low-density lipoprotein cholesterol; IFI6: interferon-alpha-inducible protein 6; IFN: interferon; MVEC: microvascular endothelial cell; FSCN1: fascin actin-binding protein 1; EMT: epithelial–mesenchymal transition; FMT: fibroblast–myofibroblast transition; GAL: glutamic acid meta-galanin; SGCA: sarcoglycan; cGAS: cyclic GMP-AMP synthase; GSEA: gene set enrichment analysis; MHC: major histocompatibility complex; ROC: receiver operating characteristic; AUC: area under the curve.
