# Supplementary material for: Relationships between systemic sclerosis and atherosclerosis: screening for mitochondria-related biomarkers
Source: Front Genet. 2024 Jul 10;15:1375331. doi: 10.3389/fgene.2024.1375331 (PMC11266065; doi:10.3389/fgene.2024.1375331)
Supplement: Supplementary file 1 [file DataSheet1.docx]

**Table S1** Identification of DEGs that had the same expression trend in GSE58095 and GSE100927

| Gene name | change | Gene name | change | Gene name | change |
| --- | --- | --- | --- | --- | --- |
| C1QTNF7 | DOWN | MFNG | UP | TMEM176B | UP |
| BEX2 | DOWN | FSCN1 | UP | DAB2 | UP |
| SCARA5 | DOWN | TMEM132A | UP | SCAMP5 | UP |
| UST | DOWN | FTL | UP | MX1 | UP |
| ANGPTL5 | DOWN | RARRES3 | UP | GZMB | UP |
| H3F3B | DOWN | COL1A2 | UP | HLA-DMA | UP |
| BARD1 | DOWN | MX2 | UP | HLA-DMB | UP |
| SGCA | DOWN | TMEM176A | UP | IFI27 | UP |
| ERRFI1 | DOWN | SHANK3 | UP | C1QC | UP |
| KLF9 | DOWN | LYL1 | UP | CD74 | UP |
| TSPAN8 | DOWN | MOXD1 | UP | FOLR2 | UP |
| RSL24D1 | DOWN | LAPTM5 | UP | CCL5 | UP |
| AGTR1 | DOWN | HCLS1 | UP | CXCL12 | UP |
| GALNTL2 | DOWN | C1QA | UP | FCN1 | UP |
| SLPI | DOWN | GNAI2 | UP | FBP1 | UP |
| SNHG5 | DOWN | NKG7 | UP | HLA-DQA1 | UP |
| PCOLCE2 | DOWN | PLEKHO2 | UP | KRT31 | UP |
| ZBTB16 | DOWN | ACP5 | UP | PFKFB4 | UP |
| ADH1A | DOWN | CSF1R | UP | RNASE1 | UP |
| THY1 | UP | COL15A1 | UP | HLA-DPA1 | UP |
| STMN2 | UP | PCOLCE | UP | FCER1G | UP |
| COL1A1 | UP | LTB | UP | CCL13 | UP |
| ISG15 | UP | SLCO2B1 | UP | HLA-DRB6 | UP |
| C2 | UP | LGMN | UP | CXCL9 | UP |
| LHFPL2 | UP | CD14 | UP | HLA-DQB1 | UP |
| BST2 | UP | EPSTI1 | UP | IGLL1 | UP |
| TIMP1 | UP | FMO3 | UP | GAL | UP |
| IFI6 | UP | STAB1 | UP | CCL18 | UP |
| C1QTNF6 | UP | KIAA1199 | UP | APOC1 | UP |
| CTSB | UP | CTSZ | UP | LUM | UP |
| SLC43A2 | UP | C1QB | UP |  |  |
| VASH1 | UP | ITGB2 | UP |  |  |
| CMTM7 | UP | FNDC1 | UP |  |  |
| SLC15A3 | UP | COTL1 | UP |  |  |
| TNC | UP | HAVCR2 | UP |  |  |
| COL6A3 | UP | COL3A1 | UP |  |  |
| MXRA5 | UP | TYROBP | UP |  |  |
| CYBA | UP | CD4 | UP |  |  |
| PSMB10 | UP | CORO1A | UP |  |  |
| SDSL | UP | MMP9 | UP |  |  |
| SLC1A3 | UP | SH2B3 | UP |  |  |

**
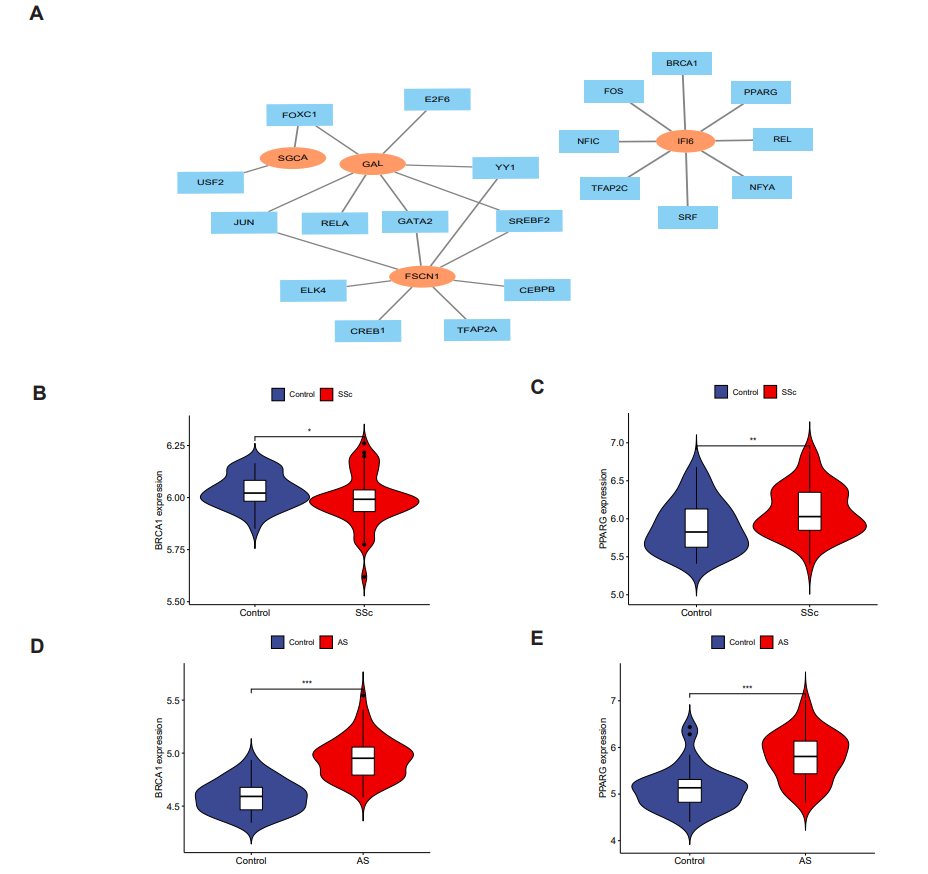
**

Fig. S1 TFs regulatory network and its expression in GSE58095 and GSE100927. (A) TFs regulatory network. TFs were marked in blue, and the hub genes were marked in yellow. (B, C, D, E) The expression level of TFs in GSE58095 and GSE100927. The comparison between the two sets of data uses the mean T test. P-value < 0.05 was considered statistically significant. SSc, systemic sclerosis; AS, atherosclerosis. *p < 0.05; **p < 0.01; ***p < 0.001.
